# Paramedics’ experiences and observations: work-related emotions and well-being resources during the initial months of the COVID-19 pandemic—a qualitative study

**DOI:** 10.1186/s12873-024-01072-0

**Published:** 2024-08-26

**Authors:** Henna Myrskykari, Hilla Nordquist

**Affiliations:** 1 Faculty of Medicine, University of Helsinki, Helsinki, Finland; 2 Emergency Medical Services, University of Turku and Turku University Hospital, Turku, Finland; 3 Department of Healthcare and Emergency Care, South-Eastern Finland University of Applied Sciences, Kotka, Finland

**Keywords:** Emergency medical services, COVID-19, Pandemics, Health personnel, Qualitative research

## Abstract

**Background:**

As first responders, paramedics are an extremely important part of the care chain. COVID-19 significantly impacted their working circumstances. We examined, according to the experiences and observations of paramedics, (1) what kinds of emotions the Emergency Medical Service (EMS) personnel experienced in their new working circumstances, and (2) what work-related factors became resources for the well-being of EMS personnel during the initial months of the COVID-19 pandemic.

**Methods:**

This qualitative study utilized reflective essay material written by experienced, advanced-level Finnish paramedics (*n* = 30). The essays used in this study were written during the fall of 2020 and reflected the period when Finland had declared a state of emergency (on 17.3.2020) and the Emergency Powers Act was implemented. The data was analyzed using an inductive thematic analysis.

**Results:**

The emotions experienced by the EMS personnel in their new working circumstances formed three themes: (1) New concerns arose that were constantly present; (2) Surviving without proper guidance; and (3) Rapidly approaching breaking point. Three themes were formed from work-related factors that were identified as resources for the well-being of the EMS personnel. These were: (1) A high level of organizational efficiency was achieved; (2) Adaptable EMS operations; and (3) Encouraging atmosphere.

**Conclusions:**

Crisis management practices should be more attentive to personnel needs, ensuring that managerial and psychological support is readily available in crisis situations. Preparedness that ensures effective organizational adaptation also supports personnel well-being during sudden changes in working circumstances.

## Background

At the onset of the COVID-19 pandemic, healthcare personnel across the globe faced unprecedented challenges. As initial responders in emergency healthcare, paramedics were quickly placed at the front lines of the pandemic, dealing with a range of emergencies in unpredictable conditions [1]. The pandemic greatly changed the everyday nature of work [2–10]. Those working on the front line were suddenly forced to adjust to personal protective equipment (PPE) requirements [9, 10] and rapidly changing instructions that caused significant adjustments to their job description [11, 12]. For instance, it has been reported that during the initial stages of the COVID-19 pandemic, Emergency Medical Services (EMS) personnel, including paramedics working in prehospital emergency care, experienced a significant increase in stress [10, 13] due to several reasons, such as the lack of protection and support, increased demands, lack of personnel, fear of exposure to COVID-19 during missions, concerns of spreading the virus to family members, and frustration over quickly changing work policies [11, 14, 15].

With the unprecedented challenges posed by the COVID-19 pandemic, some research has been directed toward identifying available resources that help in coping with such situations. For example, Sangal et al. [15] underscored the association between effective communication and reduced work stress and burnout, and emphasized the critical need for two-way communication, consistent messaging, and the strategic consolidation of information prior to its dissemination. In parallel, Dickson et al. [16] highlight the pivotal role of leadership strategies in fostering a healthful work environment. These strategies include being relationally engaging, visibly present, open, and caring for oneself and others, while embodying core values such as compassion, empathy, courage, and authenticity. Moreover, Awais et al. [14] identify essential measures to reduce mental distress and support EMS personnel’s overall well-being in pandemic conditions, such as by providing accessible mental health and peer support, ensuring a transparent information flow, and the implementation of clear, best-practice protocols and guidelines. As a lesson learned from COVID-19, Kihlström et al. (2022) add that crisis communication, flexible working conditions, compensation, and allowing for mistakes should be part of crisis management. They also emphasize the importance of psychological support for employees. [12]

Overall, the COVID-19 pandemic had a multifaceted impact on EMS personnel, highlighting the necessity for comprehensive support and resilience strategies to safeguard their well-being [11, 17, 18] alongside organizational functions [12, 19]. For example, in Finland, it has been noted in the aftermath of COVID-19 that the availability and well-being of healthcare workers are key vulnerabilities of the resilience of the Finnish health system [12]. Effective preparedness planning and organizational resilience benefit from learning from past events and gaining a deeper understanding of observations across different organizational levels [12, 19, 20]. For these reasons, it is important to study how the personnel experienced the changing working circumstances and to recognize the resources, even unexpected ones, that supported their well-being during the initial phase of the COVID-19 pandemic [12, 19].

The aim of this study was to examine the emotions experienced and the resources identified as supportive of work well-being during the initial months of the COVID-19 pandemic, from the perspective of the paramedics. Our research questions were: According to the experiences and observations of paramedics, (1) what kinds of emotions did the EMS personnel experience in the new working circumstances, and (2) what work-related factors became resources for the well-being of EMS personnel during the initial months of the COVID-19 pandemic? In this study, emotions are understood as complex responses involving psychological, physiological, and behavioral components, triggered by significant events or situations [21]. Resources are understood as physical, psychological, social, or organizational aspects of the work that help achieve work goals, reduce demands and associated costs [22].

## Materials and methods

### Setting

This qualitative study utilized reflective essay material written in the fall of 2020 by experienced, advanced-level paramedics who worked in the Finnish EMS during the early phase of the pandemic, when Finland had declared (March 17, 2020 onward) a state of emergency and implemented the Emergency Powers Act. This allowed for new rules and guidelines from the government to ensure the security of healthcare resources. Some work rules for healthcare personnel changed, and non-urgent services were limited.

### Data collection procedures

This study is part of a broader, non-project-based research initiative investigating the work well-being of paramedics from various perspectives, and the data was collected for research purposes from this standpoint. The data collection for this study was conducted at the South-Eastern Finland University of Applied Sciences as part of the Current Issues in EMS Management course. The course participants were experienced, advanced-level Finnish paramedics who were students of the master’s degree program in Development and Management of Emergency Medical Services. A similar data collection method has been utilized in other qualitative studies [for example, 23, 24].

The South-Eastern Finland University of Applied Sciences granted research permission for the data collection on August 20, 2020. The learning platform “Learn” (an adapted version of Moodle [25]) was used to gather the data. A research notice, privacy statement, and essay writing instructions were published on the platform on August 21, 2020. The paramedics were asked to write about their own experiences and observations regarding how the state of emergency impacted the work well-being of EMS personnel. They were instructed not to use references but only their own reflections. Three guiding questions were asked: “What kind of workloads did EMS personnel experience during the state of emergency?” “How has this workload differed from normal conditions?” and “What effects did this workload have on the well-being of the EMS personnel?”. The assignment did not refer solely to paramedics because the EMS field community may also include individuals with other titles (such as EMS field supervisors or firefighters performing prehospital emergency care); hence the term “EMS personnel” was used.

The essay was part of the mandatory course assignments, but submitting it for research purposes was voluntary. The paramedics were informed that their participation in the study would not affect their course evaluations. They had the freedom to decline, remove parts of, or withdraw the essay before analysis. None of the paramedics exercised these options. They were also informed that the last author removes any identifying details (such as names, places, and organizational descriptions that could reveal their workplace) before sharing the data with other, at the time unnamed, researchers. The last author (female) is a senior researcher specializing in EMS and work well-being topics, a principal lecturer of the respective course, and the head of the respective master’s program, and familiar to all of them through their studies. The paramedics were aware that the essays were graded by the last author on a pass/fail scale as part of the course assessment. However, comprehensive and well-reasoned reflections positively influenced the course grade. The evaluation was not part of this study. The paramedics had the opportunity to ask further questions about the study directly from the last author during and after the essay writing process and the course.

The paramedics wrote the essays between August 23, 2020, and November 30, 2020. Thirty-two paramedics (out of 39) returned their essays using the Learn platform during this timeframe. Thus, seven of the course completions were delayed, and the essays written later were no longer appropriate to include in the data due to the time elapsed since the initial months of the COVID-19 pandemic.

All 32 gave their informed consent for their essays to be included in the study. Essays written by paramedics who had not actively participated in EMS field work during exceptional circumstances were excluded from the material (*n* = 2), because they wrote the essay from a different perspective, as they could not reflect on their own experiences and observations. Thus, a total of 30 essays were included in the study. The total material was 106 pages long and comprised 32,621 words in Finnish.

### Study participants

Thirty advanced-level paramedics from Finland participated in this study. They all had a bachelor’s degree in emergency care or nursing with additional emergency care specialization. At the time of the study, they were pursuing their master’s studies. Thirteen of them were women, and seventeen were men. The average age of the participants was 33.5 years among women and 35.9 years among men. Women had an average of 8.7 years of work experience, and men had 8.8 years. All the participating paramedics worked in EMS in different areas across Finland (except northern Finland) during their studies and the early phase of the pandemic.

### Data analysis

The data was analyzed with a thematic analysis following the process detailed by Braun & Clarke [26]. First, the two researchers thoroughly familiarized themselves with the data, and the refined aim and research questions of the study were formulated inductively in collaboration based on the content of the data (see [26], page 84). After this, a thorough coding process was mainly carried out by the first author (female), who holds a master’s degree, is an advanced-level paramedic who worked in EMS during the pandemic, and at the time of the analysis was pursuing her doctoral studies in a different subject area related to EMS. Generating the initial codes involved making notes of interesting features of anything that stood out or seemed relevant to the research question systematically across the entire dataset. During this process, the original paragraphs and sentences were copied from the essay material into a table in Microsoft Word, with each research question in separate documents and each paragraph or sentence in its own row. The content of these data extracts was then coded in the adjacent column, carefully preserving the original content but in a more concise form. Then, the content was analyzed, and codes were combined to identify themes. After that, the authors reviewed the themes together by moving back and forth between the original material, the data in the Word documents, and the potential themes. During this process, the authors worked closely and refined the themes, allowing them to be separated and combined into new themes. For example, emotions depicting frustration and a shift to indifference formed their own theme in this kind of process. Finally, the themes were defined into main, major and minor themes and named. In the results, the main themes form the core in response to the research questions and include the most descriptions from the data. The major themes are significant but not as central as the main themes. Major themes provide additional depth and context to the results. One minor theme was formed as the analysis process progressed, and it provided valuable insights and details that deepened the response to the research question. All the coded data was utilized in the formed themes. The full content of the themes is reported in the Results section.

## Results

**Fig. 1:**
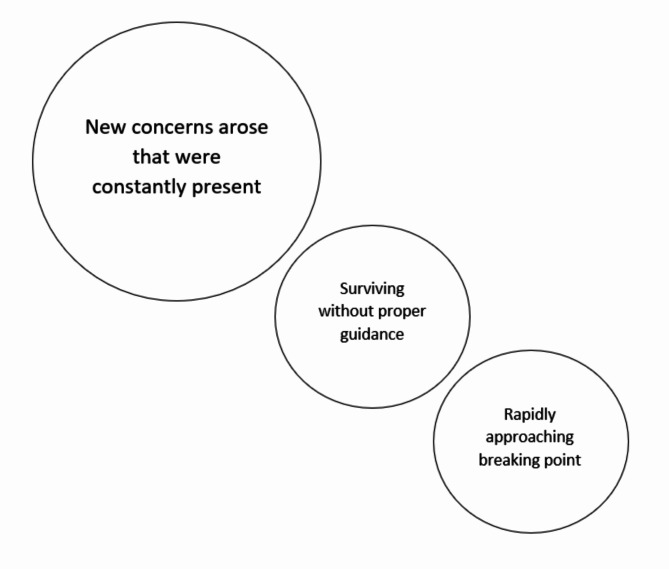
Emotions experienced by the EMS personnel in their new working circumstances

**Fig. 2:**
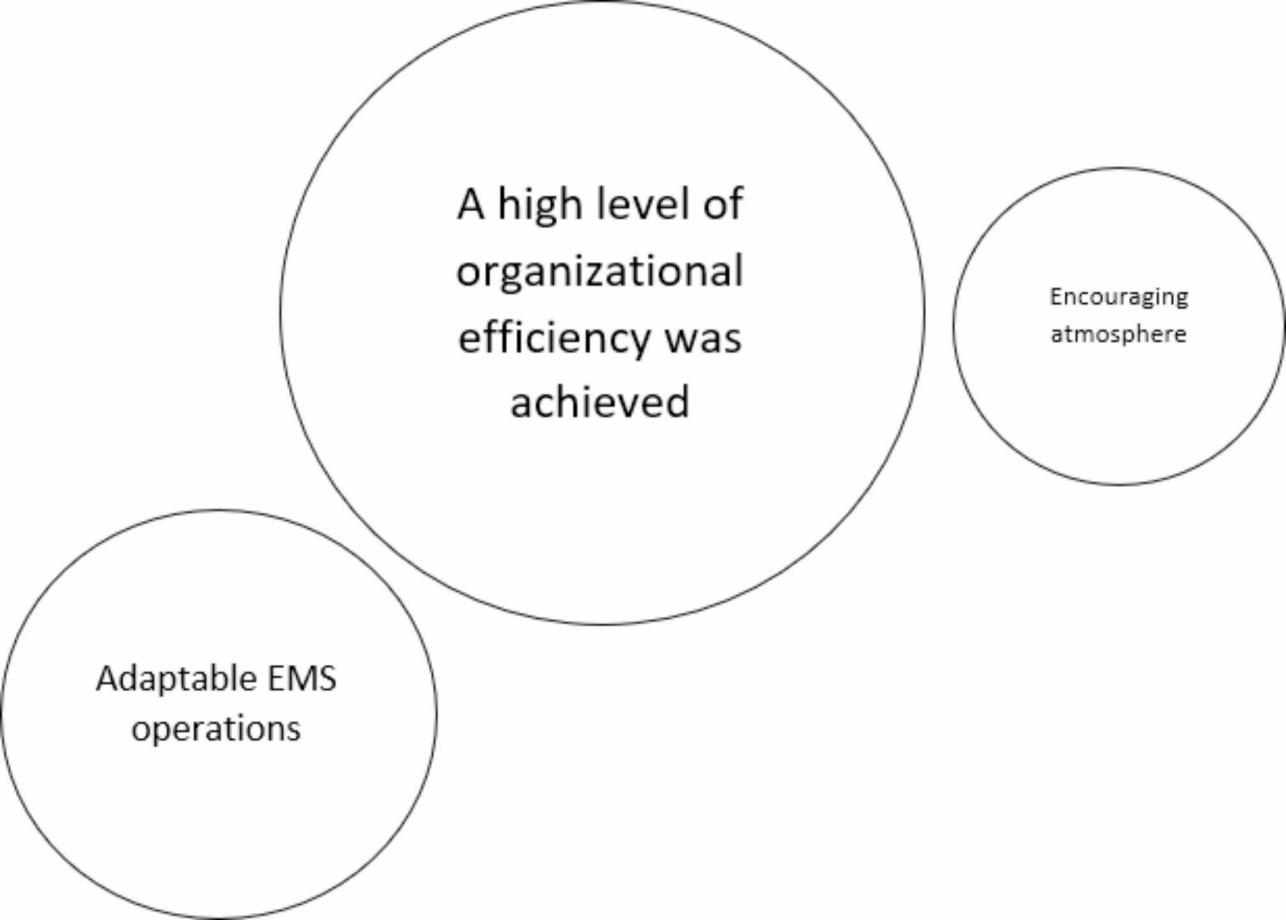
Work-related factors identified as resources for the well-being of EMS personnel

The emotions experienced by the EMS personnel in their new working circumstances formed three themes: New concerns arose that were constantly present (main theme); Surviving without proper guidance (major theme); and Rapidly approaching breaking point (major theme) (Fig. 1). Work-related factors identified as resources for the well-being of EMS personnel formed three themes: A high level of organizational efficiency was achieved (main theme); Adaptable EMS operations (major theme); and Encouraging atmosphere (minor theme) (Fig. 2).

### Main theme: New concerns arose that were constantly present

The main theme included several kinds of new concerns. In the beginning, the uncertainty about the virus raised concerns about work safety and the means to prevent the spread of the disease. The initial lack of training and routines led to uncertainty. In addition, the decrease in the number of EMS missions raised fears of units being reduced and unilateral decisions by the management to change the EMS personnel’s work responsibilities. The future was also a source of uncertainty in the early stages. For example, the transition to exceptional circumstances, concerns about management and the supervisors’ familiarity with national guidelines and lack of information related to sickness absence procedures, leave, personal career progression, and even the progress of vaccine development, all contributed to this feeling of uncertainty. The initial uncertainty was described as the most challenging phase, but the uncertainty was also described as long-lasting.

Being on the front line with an unknown, potentially dangerous, and easily transmissible virus caused daily concerns about the personnel’s own health, especially when some patients hid their symptoms. The thought of working without proper PPE was frightening. On the other hand, waiting for a patient’s test result was stressful, as it often resulted in many colleagues being quarantined. A constant concern for the health of loved ones and the fear of contracting the virus and unknowingly bringing it home or transmitting it to colleagues led the EMS personnel to change their behavior by limiting contact.

*Being part of a high-risk group*,* I often wondered*,* in the case of coronavirus*,* who would protect me and other paramedics from human vanity and selfishness [of those refusing to follow the public health guidelines]? (Participant 25)*

The EMS personnel felt a weight of responsibility to act correctly, especially from the perspective of keeping their skills up to date. The proper selection of PPE and aseptic procedures were significant sources of concern, as making mistakes was feared to lead to quarantine and increase their colleagues’ workloads. At the same time, concerns about the adequacy of PPE weighed on the personnel, and they felt pressure on this matter to avoid wastage of PPEs. The variability in the quality of PPE also caused concerns.

Concerns about acting correctly were also tied to ethical considerations and feelings of inadequacy when the personnel were unable to explain to patients why COVID-19 caused restrictions on healthcare services. The presence of students also provoked such ethical concerns. Recognizing patients’ symptoms correctly also felt distressing due to the immense responsibility. This concern was also closely tied to fear and even made some question their career choices. The EMS personnel were also worried about adequate treatment for the patients and sometimes felt that the patients were left alone at home to cope. A reduction in patient numbers in the early stages of the pandemic raised concerns about whether acutely ill individuals were seeking help. At the same time, the time taken to put on PPE stressed the personnel because it increased delays in providing care. In the early phase of the pandemic, the EMS personnel were stressed that patients were not protected from them.

*I’m vexed in the workplace. I felt it was immediately necessary to protect patients from us paramedics as well. It wasn’t specifically called for*,* mostly it felt like everyone had a strong need to protect themselves. (Participant 30)*

All these concerns caused a particularly heavy psychological burden on some personnel. They described feeling more fatigued and irritable than usual. They had to familiarize themselves with new guidelines even during their free time, which was exhausting. The situation felt unjust, and there was a looming fear of the entire healthcare system collapsing. COVID-19 was omnipresent. Even at the base station of the EMS services, movement was restricted and social distancing was mandated. Such segregation, even within the professional community, added to the strain and reduced opportunities for peer support. The EMS personnel felt isolated, and thoughts about changing professions increased.

*It was inevitable that the segregation of the work community would affect the community spirit*,* and a less able work community has a significant impact on the individual level. (Participant 8)*

### Major theme: Surviving without proper guidance

At the onset of the pandemic, the job description of the EMS personnel underwent changes, and employers could suddenly relocate them to other work. There was not always adequate support for familiarizing oneself with the new roles, leading to a feeling of loss of control. The management was described as commanding and restricting the personnel’s actions. As opportunities to influence one’s work diminished, the sense of job satisfaction and motivation decreased.

Some felt that leadership was inadequate and neglectful, especially when the leaders switched to remote work. The management did not take the situation seriously enough, leaving the EMS personnel feeling abandoned. The lack of consistent leadership and failure to listen to the personnel caused dissatisfaction and reduced occupational endurance. In addition, the reduced contact with colleagues and close ones reduced the amount of peer support. The existing models for psychological support were found to be inadequate.

Particularly in the early stages, guidelines were seen as ambiguous and deficient, causing frustration, irritation, and fear. The guidelines also changed constantly, even daily, and it was felt that the information did not flow properly from the management to the personnel. Changes in protection recommendations also led to skepticism about the correctness of the national guidance, and the lack of consistent guidelines perplexed the personnel. Internalizing the guidelines was not supported adequately, but the necessity to grasp new information was described as immense and cognitively demanding.

*At times*,* it felt like the work was a kind of survival in a jungle of changing instructions*,* one mission at a time. (Participant 11)*

### Major theme: Rapidly approaching breaking point

Risking one’s own health at work caused contentious feelings while concurrently feeling angry that management could work remotely. The arrogant behavior of people toward COVID-19 left them frustrated, while the EMS personnel had to limit their contacts and lost their annual leave. There were fears about forced labor.

Incomplete and constantly changing guidelines caused irritation and indifference, as the same tasks had to be performed with different levels of PPE within a short time. Some guidelines were difficult to comply with in practice, which was vexing.

Using a protective mask was described as distressing, especially on long and demanding missions. Communication and operation became more difficult. Some described frustration with cleaning PPE meant for single use.

Ensuring the proper implementation of a work pair’s aseptic and equipment maintenance was burdensome, and explaining and repeating guidelines was exhausting. A feeling of indifference was emphasized toward the end of a long shift.

After the initial stage, many began to slip with the PPE guidelines and found the instructions excessive. COVID-19 information transmitted by the emergency center lost its meaning, and instructions were left unheeded, as there was no energy to believe that the patient would have COVID-19, especially if only a few disease cases had been reported in their area.

It was disheartening to hear personnel being labeled as selfish for demanding higher pay during exceptional circumstances. This lack of recognition eroded professionalism and increased thoughts of changing professions.

*However*,* being a doormat and a human toilet*,* as well as a lack of appreciation*,* undermines my professionalism and the prolonged situation has led me to seriously consider a different job*,* where values other than dedication and constant flexibility carry weight. I have heard similar thoughts from other colleagues. None of us do this for money. (Participant 9)*

### Main theme: A high level of organizational efficiency was achieved

The main theme held several different efficient functions. In the early stages of the pandemic, some felt that the information flow was active. Organizations informed the EMS personnel about the disease, its spread, and its impact on the workplace and emergency care activities.

Some felt that managers were easily accessible during the pandemic, at least remotely. Some managers worked long days to be able to support their personnel.

*The response to hate and uncertainty was that one of the supervisors was always present in the morning and evening meetings. Supervisors worked long hours so as to be accessible via remote access. (Participant 26)*

The organizations took effective steps to control infections. Quick access to COVID-19 tests, clear guidelines for taking sick leave, and permission to take sick leave with a low threshold were seen as positive things. The consideration of personnel belonging to risk groups by moving them to other work tasks was also perceived as positive. In addition, efforts were made to prevent the emergence of infection chains by isolating EMS personnel in their own social facilities.

Established guidelines, especially on the correct use of protective measures, made it easier to work. Some mentioned that the guidelines were available in ambulances and on phones, allowing the protection guidelines to be checked before going on a mission.

The employers took into account the need for psychological support in a diverse manner. Some organizations provided psychological support such as peer debriefing activities, talking therapy with mental health professionals, actively inquiring about their personnel’s feelings, and training them as support workers. The pandemic situation also caused organizations to create their own standard operating models to decrease mental load.

*Fortunately*,* the problem has now been addressed actively*,* as a peer-to-peer defusing model was built up at our workplace during the crisis*,* and group defusing has started*,* the purpose of which is to lighten the work-related mental load. (Participant 3)*

### Major theme: Adaptable EMS operations

There were several different resources that clarified mission activities. The amount of protective and cleaning equipment was ramped up, and the treatment equipment was quickly updated to meet the demands brought about by the pandemic and to enable safety distances for the EMS personnel. In addition, various guidelines were amended to reduce exposure. For example, personnel on the dedicated COVID-19 ambulances were separated to work without physical contact with others, and field supervisors joined the EMS missions less often than before. Moreover, people at the scene were contacted by phone in advance to ensure that there would be no exposure risk, which also allowed other occupational safety risks to be identified. New practices resulted from the pandemic, such as cleaning communication equipment during shift changes and regularly using PPE with infected patients. All of these were seen as positive resources for efficient work.

*At the end of each shift*,* all keys*,* telephones*, etc.,* were cleaned and handed over to the next shift. This practice was not previously established in our area*,* but this will become a permanent practice in the future and is perceived by everyone in our work community as a positive thing. (Participant 10)*

Some stated that access to PPE was sufficient, especially in areas where the number of COVID-19 infections was low. PPE was upgraded to make it easier to wear. Further, organizations acquired a variety of cleaning equipment to speed up the disinfection of ambulances.

Organizations hired more employees to enable leave and the operation of dedicated COVID-19 ambulances. The overall number of ambulances was also increased. Non-urgent missions were handled through enhanced phone services, reducing the unnecessary exposure of EMS personnel to COVID-19.

*Five extra holiday substitutes were hired for EMS so that the employer could guarantee the success of agreed leave*,* even if the Emergency Preparedness Act had given them opportunities to cancel or postpone it. (Participant 12)*

### Minor theme: Encouraging atmosphere

Peer support from colleagues, a positive, comfortable, pleasant work environment, and open discussion, as well as smooth cooperation with other healthcare employees were felt to be resources for work well-being by reducing the heavy workload experienced. Due to the pandemic, the appreciation of healthcare was felt to increase slightly, which was identified as a resource.

*One factor affecting resilience in the healthcare sector is certainly that in exceptional circumstances*,* visibility and appreciation have somewhat increased. (Participant 23)*

## Discussion

This study examined, according to the experiences and observations of paramedics, (1) what kinds of emotions the Emergency Medical Service (EMS) personnel experienced in their new working circumstances, and (2) what work-related factors became resources for the well-being of EMS personnel during the initial months of the COVID-19 pandemic. Each research question was answered with three themes.

Previous studies have shown that the pandemic increased the workload of paramedics, prompting changes in their operating models and the function of EMS to align with new pandemic-related requirements [9, 27]. Initially, the paramedics in the current study described facing unclear and deficient guidelines and feeling obligated to follow instructions without adequate support to internalize them. Constantly changing instructions were linked to negative emotions in various ways. Moreover, the overwhelming flood of information was heavily connected to this, although the information flow was also perceived as a resource, especially when it was timely and well-structured. The study by Sangal et al. [15] has raised similar observations and points out the importance of paying special attention to the personnel working in the frontline, as in EMS, who might be more heavily impacted by too much information and anxiety about it. They also discovered that three factors are crucial for addressing the challenges of information overload and anxiety: consolidating information before distributing it, maintaining consistent communication, and ensuring communication is two-way. McAlearney et al. [11] found that first responders, including EMS personnel, reported frustration regarding COVID-19 information because of inconsistencies between sources, misinformation on social media, and the impact of politics. A Finnish study also recognized that health systems were not sufficiently prepared for the flood of information in the current media environment [12]. Based on these previous results and our findings, it can be concluded that proper implementation of crisis communication should be an integral part of organizations’ preparedness in the future, ensuring that communication effectively supports employee actions in real-life situations. Secondly, this topic highlights the need for precise guidelines and their implementation. With better preparedness, similar chaos could be avoided in the future [17].

Many other factors also caused changes in work. The EMS mission profile changed [3–6], where paramedics in this study saw concerns. To prevent infection risk, the number of pre-arrival calls increased [7], the duration of EMS missions increased [8, 9], and the continuous use of PPE and enhanced hygiene standards imposed additional burdens [9, 10]. In Finland, there was no preparedness for the levels of PPE usage required in the early stages of the pandemic [12]. In this study, paramedics described that working with potentially inadequate PPE caused fear and frustration, which was increased by a lack of training, causing them to feel a great deal of responsibility for acting aseptically and caring for patients correctly. Conversely, providing adequate PPE, information and training has been found to increase the willingness to work [28] and the sense of safety in working in a pandemic situation [29], meaning that the role of precise training, operating instructions and leadership in the use of PPE is emphasized [30].

The paramedics in this study described many additional new concerns in their work, affecting their lives comprehensively. It has been similarly described that the pandemic adversely affected the overall well-being of healthcare personnel [31]. The restrictions implemented also impacted their leisure time [32], and the virus caused concerns for their own and their families’ health [11, 28]. In line with this, the pandemic increased stress, burnout [10, 33], and anxiety among EMS personnel and other healthcare personnel working on the frontline [11, 14, 34, 35]. These kinds of results underscore the need for adequate guidance and support, a lack of which paramedics reported experiencing in the current study.

Personnel play a crucial role in the efficient operation of an organization and comprise the main identified resource in this study. Previous studies and summaries have highlighted that EMS personnel did not receive sufficient support during the COVID-19 pandemic [11, 14, 17, 18]. Research has also brought to light elements of adequate support related to the pandemic, such as a review by Dickson et al. [16] that presents six tentative theories for healthful leadership, all of which are intertwined with genuine encounter, preparedness, and information use. In this current study, the results showed numerous factors related to these contexts that were identified as resources, specifically underlined by elements of caring, effective operational change, knowledge-based actions, and present leadership, similarly described in a study by Eaton-Williams & Williams [18]. Moreover, the paramedics in our study highlighted the importance of encouragement and identified peer support from colleagues as a resource, which is in line with studies in the UK and Finland [12, 23, 37].

In the early stages of the pandemic, it was noted that the EMS personnel lacked adequate training to manage their mental health, and there was a significant shortage of psychosocial support measures [14], although easy access to support would have been significant [18]. In the current study, some paramedics felt that mental health support was inadequate and delayed, while others observed an increase in mental health support during the pandemic, seeing it as an incentive for organizations to develop standard operating models for mental support, for example. This awakening was identified as a resource. This is consistent, as providing psychological support to personnel has been highlighted as a core aspect of crisis management in a Finnish study assessing health system resilience related to COVID-19 [12]. In a comprehensive recommendation commentary, Isakov et al. [17] suggest developing a national strategy to improve resilience by addressing the mental health consequences of COVID-19 and other occupational stressors for EMS personnel. This concept, applicable beyond the US, supports the view that EMS organizations are becoming increasingly aware of the need to prepare for and invest in this area.

A fundamental factor likely underlying all the described emotions was that changes in the job descriptions of the EMS personnel due to the pandemic were significant and, in part, mandated from above. In this study, paramedics described feelings of concern and frustration related to these many changes and uncertainties. According to Zamoum and Gorpe (2018), efficient crisis management emphasizes the importance of respecting emotions, recognizing rights, and making appropriate decisions. Restoring trust is a significant challenge in a crisis situation, one that cannot be resolved without complete transparency and open communication [38]. This perspective is crucial to consider in planning for future preparedness. Overall, the perspective of employee rights and obligations in exceptional circumstances has been relatively under-researched, but in Australia, grounding research on this perspective has been conducted with paramedics using various approaches [39–41]. The researchers conclude that there is a lack of clarity about the concept of professional obligation, specifically regarding its boundaries, and the issue urgently needs to be addressed by developing clear guidelines that outline the obligation to respond, both in normal day-to-day operations and during exceptional circumstances [39].

Complex adaptive systems (CAS) theory recognizes that in a resilient organization, different levels adapt to changing environments [19, 20]. Barasa et al. (2018) note that planned resilience and adaptive resilience are both important [19]. Kihlström et al. (2022) note that the health system’s resilience was strengthened by a certain expectation of crisis, and they also recognized further study needs on how effectively management is responding to weak signals [12]. This could be directly related to how personnel can prepare for future changes. The results of this study revealed many negative emotions related to sudden changes, but at the same time, effective organizational adaptation was identified as a resource for the well-being of EMS personnel. Dissecting different elements of system adaptation in a crisis has been recognized as a highly necessary area for further research [20]. Kihlström et al. (2022) emphasize the importance of ensuring a healthy workforce across the entire health system. These frameworks suggest numerous potential areas for future research, which would also enhance effective preparedness [12].

### Limitations of the study

In this study, we utilized essay material written in the fall of 2020, in which experienced paramedics reflected on the early stages of the COVID-19 pandemic from a work-oriented perspective. The essays were approached inductively, meaning that they were not directly written to answer our research questions, but the aim and the research questions were shaped based on the content [26]. The essays included extensive descriptions that aligned well with the aim of this study. However, it is important to remember when interpreting the results that asking specifically about this topic, for instance, in an interview, might have yielded different descriptions. It can be assessed that the study achieved a tentative descriptive level, as the detailed examination of complex phenomena such as emotions and resources would require various methods and observations.

Although the essays were mostly profound, well-thought-out, and clearly written, their credibility [42] may be affected by the fact that several months had passed between the time the essays were written and the events described. Memories may have altered, potentially influencing the content of the writings. Diary-like material from the very onset of the pandemic might have yielded more precise data, and such a data collection method could be considered in future research on exceptional circumstances.

The credibility [42] could also have been enhanced if the paramedics who wrote the essays had commented on the results and provided additional perspectives on the material and analysis through a multi-phase data collection process. This was not deemed feasible in this study, mainly because there was a 2.5-year gap between data collection and the start of the analysis. However, this also strengthened the overall trustworthiness of the study, as it allowed the first author, who had worked in prehospital emergency care during the initial phase of the pandemic, to maintain a distance from the subject, and enabled a comparison of our own findings with previously published research that investigated the same period in different contexts. The comparison was made when writing the discussion, with the analysis itself being inductive and following the thematic analysis process described by Braun & Clarke [26].

When evaluating credibility [42], it should also be noted that the participants who wrote the essays, i.e., the data for the study, were experienced paramedics but also students and one of the researchers was their principal lecturer. This could potentially limit credibility if the students, for some reason, did not want to produce truthful content for their lecturer to read. However, this risk can be considered small because the essays’ topics did not concern the students’ academic progress, the essays’ content was quite consistent, and the results aligned with other studies. As a strength, it can be considered that the students shared their experiences without holding back, as the thoughts were not for workplace use, and they could trust the data privacy statement.

To enhance transferability [42], the context of the study was described in detail, highlighting the conditions prevailing in Finnish prehospital emergency care during the early stages of the pandemic. Moreover, including a diverse range of perspectives from paramedics working in different regions of Finland (except Northern Finland) contributes to the transferability of the study, indicating that the results may be applicable and relevant to a wider context beyond a single specific region.

Dependability [42] was reinforced by the close involvement of two researchers from different backgrounds in the analysis of the material, but a limitation is that no separate analyses were conducted. However, the original data was repeatedly revisited during the analysis, which strengthened the dependability. Moreover, the first author kept detailed notes throughout the analysis process, and the last author supervised the progress while also contributing to the analysis and reporting. The research process is also reported in detail.

## Conclusions

This study highlighted numerous, mainly negative emotions experienced by EMS personnel during the initial months of the COVID-19 pandemic due to new working circumstances. At the same time, several work-related factors were identified as resources for their well-being. The findings suggest that crisis management practices should be more attentive to personnel needs, ensuring that personnel have the necessary support, both managerial and psychological, readily available in crisis situations. Effective organizational adaptation in a crisis situation also supports personnel well-being, emphasizing the importance of effective preparedness. Future research should particularly focus on considering personnel well-being as part of organizational adaptation during exceptional circumstances and utilize these findings to enhance preparedness.

## Data Availability

The datasets generated and analyzed during the current study are not publicly available due to the inclusion of sensitive information and the extent of the informed consent provided by the participants.

## Abbreviations

CAS: Complex Adaptive Systems (theory)
COVID-19: Coronavirus Disease 2019
EMS: Emergency Medical Services
PPE: Personal Protective Equipment
UK: United Kingdom
